# Reference gene selection for molecular studies of dormancy in wild oat (*Avena fatua* L.) caryopses by RT-qPCR method

**DOI:** 10.1371/journal.pone.0192343

**Published:** 2018-02-01

**Authors:** Izabela Ruduś, Jan Kępczyński

**Affiliations:** Department of Plant Physiology and Genetic Engineering, College of Biology, Szczecin University, Szczecin, Poland; Northwestern University Feinberg School of Medicine, UNITED STATES

## Abstract

Molecular studies of primary and secondary dormancy in *Avena fatua* L., a serious weed of cereal and other crops, are intended to reveal the species-specific details of underlying molecular mechanisms which in turn may be useable in weed management. Among others, quantitative real-time PCR (RT-qPCR) data of comparative gene expression analysis may give some insight into the involvement of particular wild oat genes in dormancy release, maintenance or induction by unfavorable conditions. To assure obtaining biologically significant results using this method, the expression stability of selected candidate reference genes in different data subsets was evaluated using four statistical algorithms i.e. geNorm, NormFinder, Best Keeper and ΔCt method. Although some discrepancies in their ranking outputs were noticed, evidently two ubiquitin-conjugating enzyme homologs, *AfUBC1* and *AfUBC2*, as well as one homolog of glyceraldehyde 3-phosphate dehydrogenase *AfGAPDH1* and TATA-binding protein *AfTBP2* appeared as more stably expressed than *AfEF1a* (translation elongation factor 1α), *AfGAPDH2* or the least stable α-tubulin homolog *AfTUA1* in caryopses and seedlings of *A*. *fatua*. Gene expression analysis of a dormancy-related wild oat transcription factor *VIVIPAROUS1* (*AfVP1*) allowed for a validation of candidate reference genes performance. Based on the obtained results it can be recommended that the normalization factor calculated as a geometric mean of Cq values of *AfUBC1*, *AfUBC2* and *AfGAPDH1* would be optimal for RT-qPCR results normalization in the experiments comprising *A*. *fatua* caryopses of different dormancy status.

## Introduction

Seed dormancy is an innate seed property responsible for the temporal suppression of germination despite suitable conditions [1, 2]. Still on the mother plant, seeds acquire a state of primary dormancy which after shedding, depending on external factors (e.g. temperature, light, different chemicals), decreases gradually or is abruptly lost to allow seed germination [1]. Seeds of many plant species require variable periods of dry after-ripening or moist chilling to become non-dormant. Either in primary dormant seeds or seeds that fully or partially lost dormancy after dispersal or harvest, secondary (induced) dormancy may be imposed by unfavorable environmental cues [3, 4]. The physiological phenomenon of seed dormancy and its alterations are widely recognized to have both ecological and agricultural significance [5, 6].

*Avena fatua* L. (wild oat) is listed among the most common and economically threatening grass weed species of cereal crops all over the world [7–10]. Several traits may account for wild oat’s success as a weed, among them: high competitiveness, multiple herbicide resistance, crop mimicry (similar phenology) and varied germination strategies [8, 11]. Wild oat persistence related to diverse dormancy status of caryopses present in the soil seriously obstructs the effectiveness of weed management methods [3, 12].

Seed dormancy in *A*. *fatua* has been a matter of interest to plant physiologists since early in the XX century [13]. Several studies have been aimed at identification of genes differently expressed in dormant and non-dormant wild oat seeds [12, 14–20]. A homolog of maize transcription factor *VIVIPAROUS1* (*AfVP1*) was found to be positively correlated with the dormant phenotype of wild oat [17] and later on three VP1 interacting proteins were identified [18]. Based on the results of molecular analyses from this and other species, Shepherd et al. [21] proposed a hypothetical model of dormancy in *A*. *fatua*. More recent research has been mainly focused on physiological and biochemical aspects of hormonal regulation of *A*. *fatua* dormancy [22–25]. In their course e.g. a smoke-derived active compound—karrikinolide (KAR_1_) has been identified to act effectively in alleviation of primary dormancy in *A*. *fatua* caryopses [26–28] as well as thermodormancy [29]. The response of dormant *A*. *fatua* caryopses to KAR_1_ requires ethylene action [22] and gibberellin biosynthesis [23]. A stimulatory effect of KAR_1_ and gibberellin A_3_ on germination of dormant *A*. *fatua* caryopses can be associated with increasing activity of dehydrogenases and amylases before radicle protrusion [23, 30]. The induction of dormant *A*. *fatua* caryopses germination by KAR_1_ or GA_3_ is exerted through the regulation of ABA level in embryos and ROS-antioxidant status both in embryos and aleurone layers [24, 25]. These findings on the GA-mimicking action of KAR_1_ in dormancy release and alleviation of high temperature effects, have prompted further attempts to elucidate the underlying molecular mechanisms.

Gene expression patterns reflect the tendency of gene activity and provide an insight into gene function and gene regulatory networks. One of wide-spread methods used in gene expression analysis is real time quantitative reverse transcription-PCR (RT-qPCR) which provides means to compare the expression levels of target genes in different tissues or treatments and also to validate high-throughput gene expression profiles [31]. To obtain reliable patterns of gene expression in a given experimental system using RT-qPCR method, one must eliminate or at least reduce the influence of technical variation resulting from template input quantity and quality, yields of the extraction process, efficiency of cDNA synthesis and qPCR amplification itself [32, 33]. Currently, such a normalization of RT-qPCR data is preferentially performed using reference genes as internal controls [34]. Very often, basic metabolism genes, called house-keeping genes (HKGs), serve for that purpose, since being involved in processes essential for the survival of cells they are expected to be expressed in a constant and nonregulated manner [33]. However, the use of reference genes without prior verification of their expression stability can lead to misinterpretation of results and erroneous conclusions about real biological effects [35]. Therefore, identifying suitable candidate gene(s), minimally regulated in each specific experiment or biological setting, using statistical algorithms has become a prerequisite in qPCR analysis [32].

Recently, some commonly used reference genes for qRT-PCR have been analyzed in leaves of herbicide-resistant wild oat biotypes [10]. However, to date, no endogenous control genes for RTq-PCR have been identified for other treatments or developmental stages in *A*. *fatua*. Taking into account that seeds show distinct transcriptomes compared with the vegetative tissues [36–40], there is a need for re-validation of reference genes for analyses dedicated to wild oat caryopses. Thus, in the present study, a qRT-PCR protocol based on SYBR reagent was used for the identification of genes with minimal expression variation during primary dormancy release and thermodormancy induction in *A*. *fatua* caryopses. Seven homologs of genes traditionally used as internal controls [33, 41] were selected as candidate reference genes for evaluation, including translation elongation factor (*EF1α*), α-tubulin (*TUA*) and TATA-binding protein (*TBP*) as well as two homologs of glyceraldehyde 3-phosphate dehydrogenase (*GAPDH*) and ubiquitin-conjugating enzyme (*UBC*). The expression stability of these genes was assessed by four different statistical algorithms to select the most suitable internal control. The validation of the optimal reference gene was performed on dormancy-related *AfVP1* gene. Our work was aimed to assist future studies on molecular basis of seed dormancy induction, maintenance, and release in wild oat as a contribution to the on-going research focused on characterization of functional networks of seed dormancy-associated genes.

## Material and methods

### Ethics statement

Before entering the crop field, an oral permission for the collection of caryopses was obtained from the land owner. No other permits or approvals for the collection of plant material used in the studies were required because *A*. *fatua* is not endangered or protected species in Poland. It is not mentioned in any of the relevant documents [42–44]. On the contrary, as a dangerous weed species, it is subject to different weed control programs, usually including herbicide application.

### Plant material

Ripe caryopses of *Avena fatua* L. were obtained from plants naturally growing in Poland. Spikelets were collected in crop fields near Szczecin in July 2011. After collection, they were dried at room temperature for 7 days to a constant moisture content (~ 11%). Afterwards, one part of harvested spikelets was immediately stored at -20 °C to maintain primary dormancy of caryopses, the other part was subjected to after-ripening, i.e. dry-storage at 25 °C for six months. Thus released from dormancy caryopses were then also kept at -20 °C. In the experiments, only dehulled caryopses were used so both lemma and palea were removed by hand.

The expression stability of putative reference genes was analyzed in primary dormant (PD) and non-dormant (ND) *A*. *fatua* caryopses, either dry or subjected to different temperatures or application of plant growth regulators during imbibition. For comparison with the vegetative tissues, also samples of leaves and roots of eight day old seedlings were examined.

PD or ND caryopses, 25 per sample, were incubated in darkness, in 6-cm Petri dishes on one layer of filter paper (Whatman No. 1) moistened with 1.5 mL deionized water or a solution of applied plant growth regulator, karrikinolide KAR_1_ (3x10^-9^ M) or gibberellin A_3_ (10^−5^ M). The temperatures of incubation were 20, 30 or 35 °C, of which the latter two were found as supraoptimal temperatures (SOT) inducing thermodormancy in PD and/or ND caryopses. Samples were collected at three or four time points (hour 8, 24, 36 for all combinations and 41 or 96 for selected treatments) during imbibition before the completion of germination that is before radicle protrusion through colleorhiza.

In total, samples of eleven treatments of ND and seventeen treatments of PD caryopses (differing in incubation time, temperature, solution as indicated in S1 Table) as well as two types ofvegetative organs (leaf and root) were obtained, each of them in three biological replicates. All collected samples were snap-frozen in liquid nitrogen and stored at -80 °C until used.

### Total RNA preparation, quality control and cDNA synthesis

Total RNA from caryopses was extracted according to the protocol described by Oñate-Sánchez and Vicente-Carbajosa [45] for seeds and tissues with a high content of polysaccharides, with some modifications. Each sample of plant material comprised 25 caryopses which were ground in a mortar using liquid nitrogen. Ca. 80 mg of powdered tissue was mixed with 1 ml of the extraction buffer (0.2 M Tris pH:8, 0.4 M LiCl, 25 mM EDTA, 1% SDS), 0.5 ml chloroform and 0.1 ml Plant RNA Isolation Aid (Ambion). The extraction mixture was incubated on ice for 10 min and centrifuged (14,000 rpm, 15 min, 4°C). RNA was then purified from the lysate with water-saturated acidic phenol (pH:4.5) and chloroform. Afterwards, RNA was precipitated overnight at 4 °C from aqueous phase through addition of 1/2 volume of 5 M LiCl and spinned for 30 min at 4 °C. The pellet was then washed with 75% cold ethanol.

The isolation of total RNA from seedlings (leaf/root separately) was conducted from 100 mg of frozen tissue homogenized in 1 ml Tri Reagent solution (Sigma-Aldrich). After homogenization, samples were incubated at room temperature for 5 min, vortexed and centrifuged (14,000 rpm, 10 min, 4°C) to remove the insoluble material. For phase separation, 100 μl of BCP (1-bromo-3-chloropropane) was added to the supernatant, mixed by inversion and kept at room temperature for 5 min followed by centrifugation (14,000 rpm, 15 min, 4°C). RNA from the aqueous phase was precipitated with isopropanol and washed with ethanol.

Total RNA, isolated either from caryopses or seedlings, was suspended in 25 μl of RNase-free water and subjected to quantitative and qualitative control. The RNA concentration of each sample was estimated using a BioSpec-Nano micro-volume UV-Vis Spectrophotometer (Shimadzu Scientific Instruments) and RNA integrity was assessed by 2% agarose gel electrophoresis through visualisation of the two ribosomal subunits. Potential genomic DNA contamination was eliminated by DNase I (Ambion) treatment of all RNA samples. They were suspended in 25 μl nuclease-free water and their concentration, purity and integrity were re-checked. Only the RNA samples with A260/A280 ratios between 1.8 and 2.1 and A260/A230 ratios greater than 2.0 were further used. First-strand cDNA of each sample was synthetized from 1 μg of DNase-treated total RNA in a 20 μl reaction volume using the High-Capacity cDNA Reverse Transcription Kit (Thermo Fisher) according to the manufacturer’s protocol. cDNA samples were ten times diluted with nuclease-free water and used for RT-qPCR.

### Candidate reference gene selection and primer design

Candidate genes were selected based on their previous description as good plant internal control genes for qRT-PCR analysis, including seeds, in a number of species [10, 40, 41, 46–48]. Homologous sequences of these selected genes were retrieved from our *A*. *fatua* RNA-seq assembled transcriptome dataset (S1 File) by sequence search using cDNA sequences of homologous genes identified in different species of *Poaceae* family performed in Geneious R6 software (Biomatters Ltd.) (S2 Table). Strict parameters were used to identify the BLASTn hit with greatest homology and included: the full/partial transcript being >400 bp in length; an E-score <1e^-20^, and greatest homology the query sequence according to the Bit score and pairwise identities for the transcript and deduced amino acid sequences.

The qRT-PCR primers for reference gene candidates, except for *AfTBP2*, and also for the target gene *AfVP1*, were designed with Primer3: WWW primer tool [49], using the following parameters: amplicon length around100 bp; primer size between 18 and 22 bp; melting temperature (Tm) between 57 and 61°C; GC content between 40 and 60%. Amplification primers used for *TBP* were adopted from the published sequence data of Wrzesińska et al. [10]. The sequences of qRT-PCR primers with the characteristics of their corresponding amplicons are listed in Table 1 and S3 Table.

**Table 1:** Gene-specific primers and amplicon characteristics of candidate reference genes and a target gene used in RT-qPCR analysis.

Gene name	Target sequence	Primer sequence (5’ → 3’) (forward/reverse)	Amplicon length (bp)	Tm (°C)^a^	Amplification efficiency^b^	R^2^
*AfEF1a*	CL3197 contig1	CACCCCCAAGTACTCCAAG GACAAATGGGACCTTGTCAG	100	80.53	1.859	0.99992
*AfGAPDH1*	Unigene 36693	TTCTTCCTGAGTTGAACGGC ATGCAGCCTTCTCGATTCTG	102	80.67	1.879	0.99997
*AfGAPDH2*	Unigene 36695	GAATACACCTCTGACATCACCA CTCAACAATGCCAAACTTGTCA	102	79.93	1.883	0.99994
*AfTBP2*	CL4896 contig1	TGAGCCAGAACTTTTTCCTG CTTTGCACCGGTCAAGACA	100	78.14	1.881	0.99995
*AfTUA1*	Uni36141	GTGCCTACCGTCAGCTTTT CGATCTCCTTTCCAACAGTGT	102	83.94	1.868	0.99997
*AfUBC1*	Uni2728	ACAGTAACGGGAGCATATGTC GGTCCGTTAGCAGAGAACAG	102	78.43	1.867	0.99994
*AfUBC2*	CL162 contig2	GGACATTTTCGAGTGGCAAT GGGTAGTCAGAGGGCAACTG	99	80.81	1.860	0.99995
*AfVP1*	CL7717 contig1	TGACTTTATGTTCGCGGAAG AGTTGGAGGAGGATGAGGAG	101	82.62	1.807	0.99989

^a^ The amplicon melting temperature calculated by StepOne software

^b^ The amplification efficiency calculated by LinReg software

### RT-qPCR conditions and primer specificity

Real-time reactions were performed using SYBR Select Master Mix (Applied Biosystems) as follows: 1 μL of cDNA diluted 10 times, 5 μL of the mix and 0.2 μM of each primer, in a final volume of 10 μL. The StepOne system (Applied Biosystems) was employed and PCR cycling consisted of the following steps: initial 2 min at 95 °C for pre-denaturation, followed by 40 cycles of 15 s at 95 °C for denaturation and 1 min at 60 °C for annealing and extension. Afterwards, the final extension was performed at 60 °C for 1 min and was followed by melting curve analysis using default parameters in order to verify the PCR specificity by constant increase in temperature from 60 °C to 95 °C, at increments of 0.5 °C. Three biological replicates per treatment/organ type were analyzed with each PCR reaction done in triplicate.

### Primer amplification efficiency calculation and Cq determination

Efficiency of each primer pair and the quantification cycle (Cq) determination were done by LinRegPCR method [50]. Cq value is the amplification cycle number at which the fluorescence from amplification reaches a set threshold of signal level [51]. Raw fluorescence data generated with the StepOne software (Applied Biosystems) was used for these calculations. LinRegPCR calculates individual efficiency values for each RT-qPCR reaction and averages them to obtain mean efficiency value for each gene. After running the LinReg algorithm, Cq values were transferred as a Microsoft Excel file (Microsoft, Redmond, WA) for further gene expression stability analysis.

### Analysis of stability of candidate reference genes

Expression stability of the seven potential internal control genes across all treatments and samples in total and in subsets were determined using four statistical algorithms: geNorm [52], NormFinder [53], BestKeeper [54] and the comparative ΔCt method [55] following developer’s instructions. The Cq values of all reference genes used in the geNorm and NormFinder were converted into relative quantities by ΔCq method based on the efficiencies of primers. The geNorm algorithm was used to rank the reference genes by calculating their expression stability value M determined as the standard deviation of the logarithmically transformed expression ratios of a particular gene and all other candidate genes, with 1.5 as a recommended cut-off threshold value. The lower M value was attributed to the particular gene, the more stable it was considered. Additionally, using the geNorm software the pairwise variations (Vn/Vn+1) was calculated in order to determine the minimum number of reference genes for optimal normalization. An expression stability value (M) for each candidate reference was calculated with NormFinder software, based on the inter and intragroup variance estimation approach. Three main measures offered by BestKeeper software: the standard deviation (SD) of the Cq of all samples for each analyzed gene, the coefficient of variation (CV) of a potential reference gene and the correlation coefficient with the BestKeeper index (r) were used to evaluate gene expression stability. Since the BestKeeper program does not indicate which of them is more reliable nor has more weight to select the best reference gene, we used the approach described by Olias et al. [56]. First, each candidate reference gene was evaluated for the three measures (SD, CV and r) separately, then the mean of the rankings was calculated to determine the final rank of each gene. In the comparative ΔCt method, candidate reference genes were ranked due to the mean standard deviation of ΔCt in analyzed samples.

### Validation of reference genes

To validate the choice of the optimal reference genes in our experimental system, their performance as single or combined normalization factors in qRT-PCR analysis of a target gene was assessed. The expression levels of *AfVP1* gene were normalized by the most stable reference genes (according to the expression stability rankings provided by four different statistical algorithms). Samples were collected from ND and PD caryopses, either dry or incubated in water at 20 °C for 8, 24 or 36 hours. The relative expression ratios were calculated using the efficiency corrected model [54]. The final value of relative quantification was described as fold change of gene expression in the test samples (ND caryopses) compared to control (PD caryopses) which served as a calibrator with set value of 1 at each time-point. Data were expressed as mean ± SD of three biological replicates for each treatment.

## Results

### Candidate reference gene selection and sequences identification

The selection of genes which could serve as reference genes for normalization in qRT-PCR gene expression analysis in *A*. *fatua* was based on literature recommendations. Since only very limited wild oat genome sequence information was available, the *A*. *fatua* transcriptome dataset was queried for the chosen (*EF1α*, *GAPDH*, *TUA*, *UBC*) candidate genes using the publicly available gene sequences from *Avena sativa*, *Hordeum vulgare* and *Brachypodium distachyon*, all belonging to *Poaceae* family. The longest homologous sequences of *A*. *fatua* showing the highest similarity to the query sequences were selected as targets for qRT-PCR analysis (Table 1, S2 Table). There were, however, also other sequences retrieved for each of the candidate gene indicating the existence of multigene families. In two cases, namely *GAPDH* and *UBC* probable gene families, two paralogs were appointed as candidate reference genes and their expression stability was subsequently assessed.

Whenever possible, the retrieved sequences were compared with partial coding sequences of *A*. *fatua* recently submitted to NCBI database. The results of the pairwise alignment of these transcripts revealed some sequence discrepancies (Table 2). Homolog *AfEF1a* (CL3197 contig1) represented nearly complete coding sequence missing only 7 codons at 3’ end, whereas its counterpart from GenBank (KT153026) was shorter. There was a 94.6% pairwise identity between them. Two homologues transcripts, *AfGAPDH1* (Unigene36693) and *AfGAPDH2* (Unigene36695) comprised full or nearly full (only one codon missing) coding sequences if compared to the reference *GAPDH* sequences from *H*. *vulgare*. They showed 99.3% and 83.2% identity with the partial *AfGAPDH* cds (KT153027), respectively. Out of several retrieved homologous transcripts of *TUA*, the longest was Unigene36141 designated as *AfTUA1* and further analyzed for its expression stability. *AfTUA1* showed only 79.3% pairwise identity with the partial *AfTUA* transcript available in NCBI database (KT153029). Higher identity of 96.7% was observed between KT153029 and Unigene2539, confirming the presence of the second *AfTUA* homolog.

**Table 2:** Comparison of partial coding *A*. *fatua* sequences with transcripts retrived from RNA-seq assembled dataset. Global pairwise alignment with free end gaps was conducted using Geneious software (Cost matrix: 65% similarity 5.0/-4.0; gap open penalty: 12; gap extension penalty:3).

Gene symbol	Partial coding A. fatua sequences^a^		A. fatua transcripts^b^		*A*. *fatua* putative gene homolog	Pairwise identity (%)
	Accession numbers	Sequence length (bp)	Transcript symbol	Transcript length (bp)		
*EF1a*	KT153026	834	CL3197 contig1	1341	*AfEF1a*	94.6
*GAPDH*	KT153027	849	Unigene 36693	1011	*AfGAPDH1*	99.3
			Unigene 36695	1008	*AfGAPDH2*	83.2
*TBP*	KT153028	497	CL3311 contig2	570	*AfTBP1*	83.1
			CL4896 contig1	351	*AfTBP2*	97.5
*TUA*	KT153029	792	Unigene 36141	1218	*AfTUA1*	79.3
			Unigene 2539	957	*AfTUA2*	96.7

^a^ nucleotide sequences retrived from GeneBank database

^b^ nucleotide sequences retrived from RNA-seq assembled dataset

The use of a partial *AfTBP* sequence (KT153028) in the search of homologs in our NGS transcriptome dataset revealed the presence of even eight *AfTBP* paralogs (S2 Table). The KT153028 query had the highest identity of 97.5% with CL4896 contig1 designated *AfTBP2* due to its similarity to *Oryza sativa TBP2* gene (AF464907), while 83.1% with the longest transcript CL3311 contig2, designated *AfTBP1* (Table 3). Only the expression stability of *AfTBP2* was further analyzed for the sake of comparison with previous results of Wrzesińska et al. [10]. The sequence of the target gene *AfVP1* (AJ001140) had 98.6% pairwise identity with the transcript found in the NGS transcriptome dataset (S2 Table).

**Table 3:** Sample datasets for statistical analysis of expression stability of candidate reference genes in *A*. *fatua*. Symbols of sample groups refer to S1 Table. For each sample set, a count (N) of sample combinations included in analysis are given.

Sample set			
Symbol	Description	Sample groups included	N
Total	The samples of all treatments and organ types	All samples	30
NPC	Nondormant and primary dormant caryopses, dry or incubated in water at 20 °C	dN, N-20H, dP, P-20H	9
NPC+SOT	Nondormant and primary dormant caryopses, dry or incubated in water at 20 or 30 °C	dN, N-20H, N-30H, dP, P-20H, P-30H	16
NC+S	Nondormant caryopses, dry or incubated in water at 20 °C as well as leaf and root samples	dN, N-20H, SL, SR	6
NC+SOT	Nondormant caryopses, dry or incubated in water at 20, 30 or 35 °C	dN, N-20H, N-30H, N-35H	11
PC+KG	Primary dormant caryopses, dry or incubated at 20 °C in water or the solutions of KAR_1_ or GA_3_	dP, P-20H, P-20K, P-20G	13

### Primer specificity, amplification efficiency and gene expression profiles

The gene specific primers were designed to accurately amplify the candidate reference genes using qRT-PCR. The melting temperatures (Tm) of all PCR products ranged from 78.14 °C for *AfTBP2* to 83.94 °C for *AfTUA1* (Table 1). All the primer pairs amplified a single PCR product of the expected size, which appeared as one peak on the melting curve (S1 Fig).

According to Taylor et al. [57], amplification efficiencies within the range of 1.8–2.0 can be considered ideal for qRT-PCR and R^2^ values greater than 0.98 indicate that the efficiencies are reliably determined. In our analyses, the PCR efficiency of each of the qRT-PCR primer pairs met these standards. All primer pairs had mean efficiencies between 1.807 and 1.883, therefore sufficient for qRT-PCR analysis (Table 1). Also, very high values of the coefficents of determination exceeding 0.999, implied high accuracy of efficiency estimation.

**Fig 1:**
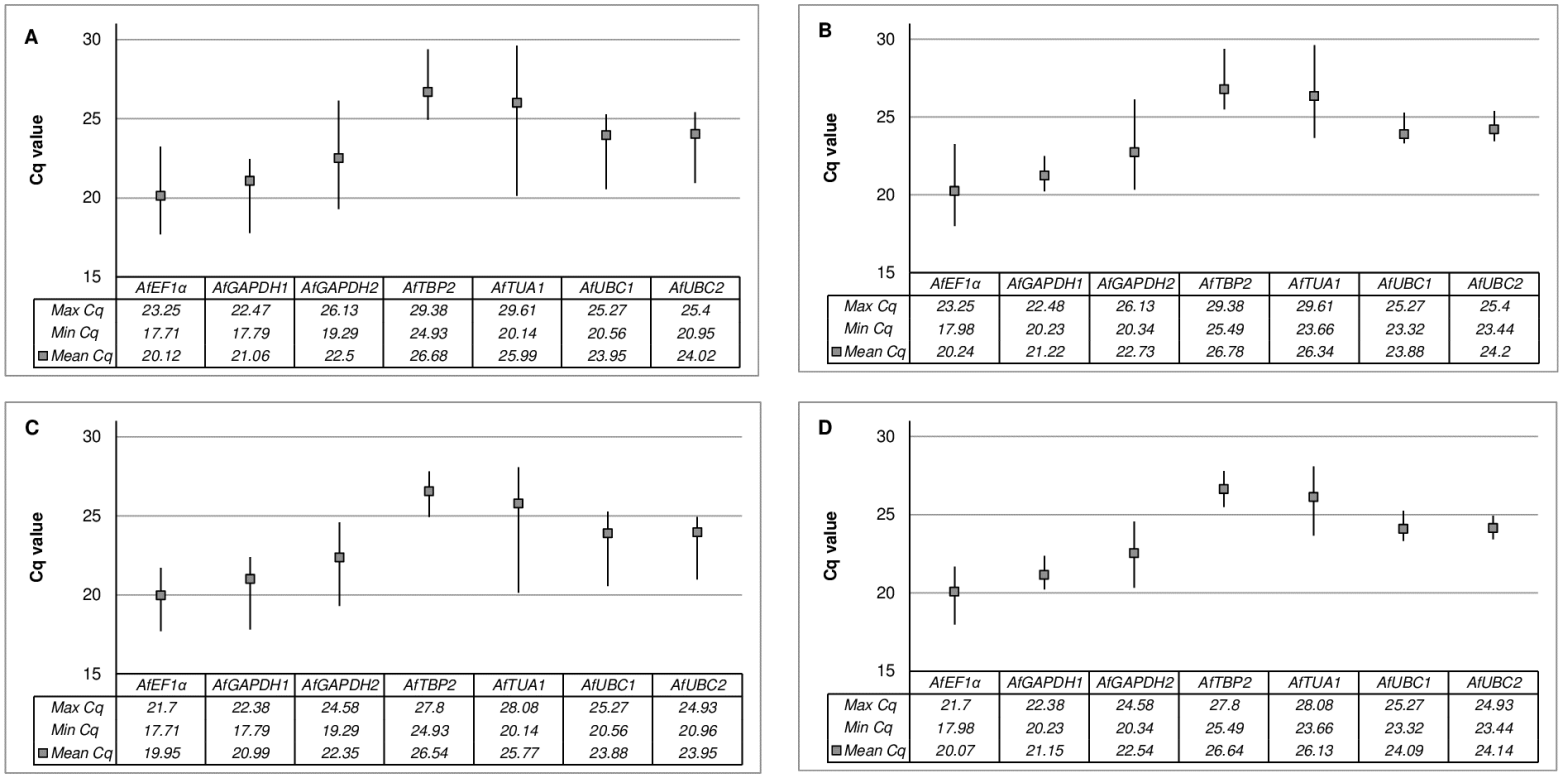
Distribution of candidate reference gene Cq values. The data represent raw qRT-PCR values obtained for different sample sets: Total (A), dry and imbibed caryopses (B), only imbibed caryopses with (C) or devoid of seedling organs (D). Whiskers indicate the Cq range. The mean Cq value is indicated by a filled square.

Since all qRT-PCR reactions were performed with an equivalent amount of template cDNA, transcript abundance of these genes in different samples was estimated by direct comparison of Cq values (Fig 1). Over all samples, the Cq values of the seven candidate reference genes ranged from 17.71 to 29.61 (Fig 1A). Among them, *AfEF1α* was the most abundantly expressed in all of the samples (mean Cq ± SD = 20.12 ± 1.21) followed by two *AfGAPDH* paralogs, *AfGAPDH1* (mean Cq ± SD = 21.06 ± 0.76) and *AfGAPDH2* (mean Cq ± SD = 22.53 ± 1.51), whereas *AfTBP2* had the lowest transcription level (mean Cq ± SD = 26.68 ± 0.80). *AfTUA1* was close in Cq value but substantially differed in SD value (mean Cq ± SD = 25.99 ± 1.78) in comparison to *AfTBP2*. The expression of *AfTUA1* was the most variable among all candidates tested, with the widest Cq range of 9.46 cycles. Lower discrepancies in Cq values were detected for *AfEF1α* and *AfGAPDH2* (5.64 and 6.84 cycles respectively). The variability of four other genes`expression levels were quite even, showing differences of ca. 4.5 cycle. A considerable contribution to the differences in expression levels of most analyzed genes came from seedling samples (Fig 1B). As soon as they were omitted from the calculations, a substantial decrease (by more than 1 cycle) in Cq range was observed, especially for *AfGAPDH1*, *AfTUA1*, *AfUBC1* and *AfUBC2*. Only *AfEF1α* and *AfTBP2* remained then unaffected. In comparison, when Cq value from dry caryopses where excluded, a significant decrease in expression level range was observed for *AfEF1α*, *AfTBP2* and also *AfTUA1*, but not for *AfGAPDH1*, *AfUBC1* and *AfUBC2* (Fig 1C). If only Cq values from imbibed caryopses were analyzed, both the Cq range and SD achieved minimal values (Fig 1D). Either standard deviation or the range of Cq values can reveal the expression stability of candidate reference genes, however more robust stability ranking was obtained using the four different computational programs.

### Expression stability of candidate reference genes

In order to perform the comprehensive expression analysis of candidate reference genes and choose an appropriate normalizing factor for different experiments, the statistical analyses were not only conducted in the overall dataset comprising all biological samples but also in five subsets (sample groups) distinguished due to the initial dormancy status of caryopses and imbibition conditions (Table 3). The Cq values for each candidate reference gene were used for stability comparison.

**Fig 2:**
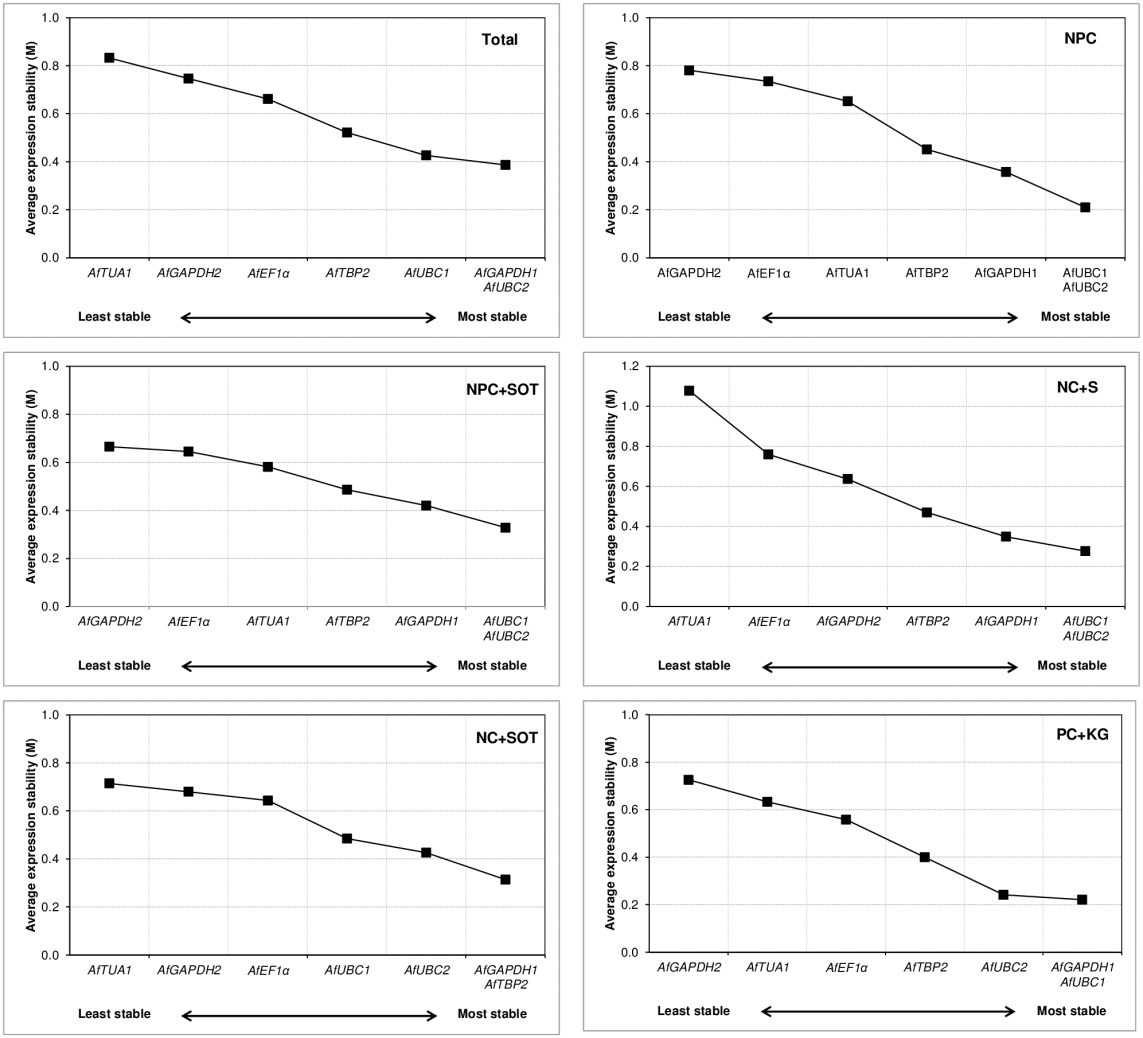
Gene expression stability and ranking of seven candidate reference genes based on geNorm algorithm. Each panel refers to a distinct dataset described in Table 3. Average stability measure (M) was calculated following stepwise exclusion of the least stable gene. Lower M values indicate more stable gene expression.

In geNorm analysis none of the candidate reference genes had M value higher than 1.5 thus exhibiting a relative stability (Fig 2). When the results from all samples of *A*. *fatua* caryopses and seedlings were combined (Total dataset), *AfGAPDH1* and *AfUBC2* had the highest expression stability with M value of 0.387, closely followed by *AfUBC1* (M = 0.427). In most data subsets, these three reference genes were also ranked at the top, though flipping sometimes in their positions. Only in the subset NC+SOT, a sample group comprising ND caryopses subjected to SOT treatments, the pair of highest stability indicated by geNorm was *AfGAPDH1* and *AfTBP2* (M = 0.314), while *AfUBC2* and *AfUBC1* occupied the 3^rd^ (M = 0.426) and 4^th^ (M = 0.485) position respectively. Two reference genes were indicated by geNorm analysis as the most unstable, either *AfTUA1* or *AfGAPDH2*, depending on the data subset.

**Fig 3:**
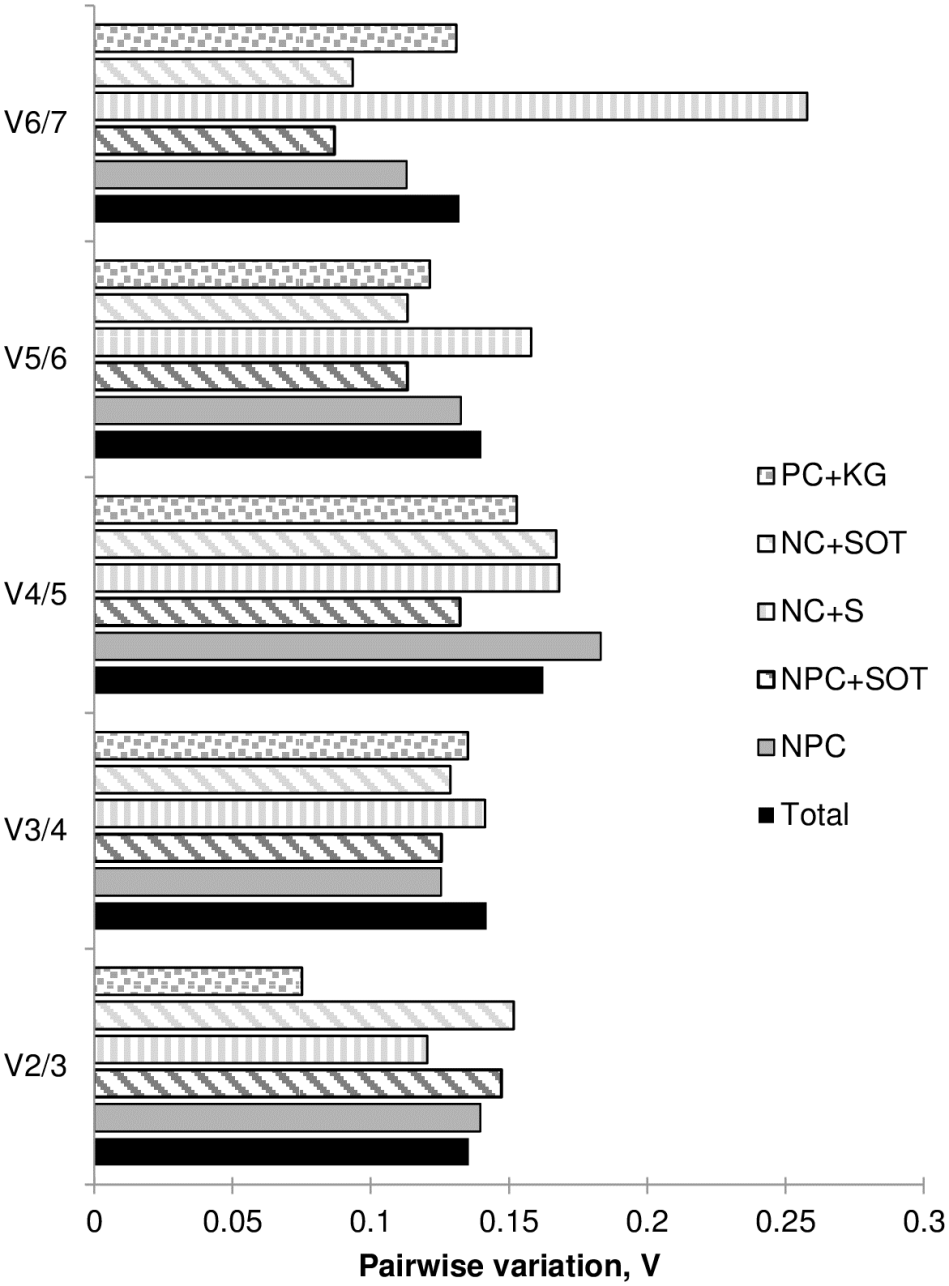
Pairwise variation (V) analyses of the candidate reference genes. The pairwise variation (V_n/n+1_) was analyzed for the normalization factors NFn and NFn+1 by the geNorm program to determine the optimal number of reference genes for accurate normalization. The cutoff value was proposed to be 0.15, below which the inclusion of an additional reference gene is not necessary. For dataset symbols, refer to Table 3.

The results of the pairwise variation measure showed that generally in all experimental sample groups, the V2/3 value was very close to 0.15 (Fig 3) which is a commonly accepted cut-off threshold [52]. In the Total dataset and two data subsets (NC+S, PC+KG), the pairwise variation increased for V3/4 and V4/5, confirming that the two top-ranked candidate reference genes would be sufficient as the normalization factor for these sample groups. For three other data subsets i.e. NPC, NPC+SOT and NC+SOT, the variation value V3/4 was lower than V2/3, which was nearly as high or even slightly exceeding the 0.15 threshold. Such results suggested that in their case three rather than two top ranked genes should be included for calculation of a reliable normalization factor.

In the Total dataset, *AfUBC1* (M = 0.286) was appointed as the most stable gene by NormFinder (Table 4). Next two positions in the stability ranking were assigned to *AfUBC2* (M = 0.314) and *AfTBP2* (M = 0.327). So, two out of three geNORM top-ranked genes appeared as highly stable in NormFinder algorithm. Unexpectedly, *AfGAPDH1* was ranked only at the 5^th^ position, but together with *AfGAPDH2*, gave the optimum combination. Interestingly, both *AfGAPDH* paralogs were individually assessed as rather unstable, and yet, when paired, exhibited a low variation value of 0.155. In the data subsets, either *AfTBP2* (NPC, NC+S, PC+KG) or *AfUBC2* (NPC+SOT, NC+SOT) were indicated by NormFinder as the most stable (M value between 0.2 and 0.3). Thus *AfTBP2* and two *AfUBC* paralogs appeared as the three top-ranked in stability by NormFinder, although in PC+KG data subset, *AfUBC2* was assigned only 6^th^ position. Similarly as in geNorm analysis, usually *AfTUA1* and *AfGAPDH2* were at the bottom of stability rankings in NormFinder.

**Table 4:** Candidate genes expression stability value (M) of single and best combination of two genes estimated by NormFinder algorithm in different datasets. For dataset symbols, refer to Table 3.

Sample set	Candidate reference gene														Best combination of two genes	
	*AfEF1a*		*AfGAPDH1*		*AfGAPDH2*		*AfTBP2*		*AfTUA1*		*AfUBC1*		*AfUBC2*			
	M	Rank	M	Rank	M	Rank	M	Rank	M	Rank	M	Rank	M	Rank		M
Total	0.360	4	0.364	5	0.432	7	0.327	3	0.411	6	0.286	1	0.314	2	*AfGAPDH1*, *AfGAPDH2*	0.155
NPC	0.387	5	0.373	4	0.414	6	0.273	1	0.423	7	0.312	3	0.305	2	*AfEF1a*, *AfGAPDH1*	0.121
NPC+SOT	0.340	6	0.291	4	0.330	5	0.278	3	0.353	7	0.276	2	0.255	1	*AfEF1a*, *AfGAPDH1*	0.136
NC+S	0.611	6	0.440	5	0.358	3	0.304	1	0.949	7	0.381	4	0.346	2	*AfGAPDH1*, *AfUBC1*	0.143
NC+SOT	0.354	6	0.348	5	0.346	4	0.324	3	0.370	7	0.240	2	0.221	1	*AfEF1a*, *AfTBP*	0.094
PC+KG	0.291	2	0.339	5	0.547	7	0.217	1	0.311	4	0.297	3	0.360	6	*AfTUA1*, *AfUBC1*	0.107

The stability rankings for candidate reference genes in different data subsets were calculated on the basis of three measures generated by BestKeeper program (SD, CV and r) (Table 5). Similarly to geNorm, in the Total dataset and most of the subsets, the genes of highest stability assessed by BestKeeper, were *AfUBC2*, *AfUBC1* and *AfGAPDH1*. Some discrepancies between BestKeeper (Table 5) and geNorm (Fig 2) concerned stability evaluation of *AfGAPDH1* and *AfTBP2* in two data subsets comprising only ND caryopses. The ranks of these two genes assigned by BestKeeper, 1^st^ place in NC+S and 3^rd^ place in NC+SOT for *AfTBP2*, while 4^th^ and 5^th^ for *AfGAPDH1*, respectively, repeated the results from NormFinder (Table 4). The least stable genes assessed by BestKeeper were also *AfTUA1* and *AfGAPDH2*.

**Table 5:** Ranking of candidate reference genes according to the BestKeeper analysis of their expression stability. For dataset symbols, refer to Table 3.

Sample set		Candidate reference genes													
		*AfEF1A*		*AfGAPDH1*		*AfGAPDH2*		*AfTBP2*		*AfTUA1*		*AfUBC1*		*AfUBC2*	
		SV	Rank	SV	Rank	SV	Rank	SV	Rank	SV	Rank	SV	Rank	SV	Rank
Total	SD	0.99	5	0.46	1	1.25	7	0.59	4	1.22	6	0.58	3	0.47	2
	CV	4.89	6	2.19	2	5.56	7	2.21	3	4.70	5	2.41	4	1.98	1
	r	0.881	4	0.793	7	0.930	2	0.820	6	0.966	1	0.882	3	0.839	5
	Mean of sub-ranks		5.00		3.33*		5.33		4.00		4.33		3.33*		2.67
	BK ranking	**5**		**2**		**6**		**3**		**4**		**2**		**1**	
NPC	SD	1.06	3	0.39	1	1.12	4	0.69	2	1.20	5	0.39	1	0.39	1
	CV	5.18	7	1.82	3	4.82	6	2.52	4	4.44	5	1.64	2	1.60	1
	r	0.979	1	0.702	7	0.962	2	0.882	5	0.929	3	0.920	4	0.891	6
	Mean of sub-ranks		3.67*		3.67*		4.00		3.67*		4.33		2.33		2.67
	BK ranking	**3**		**3**		**4**		**3**		**5**		**1**		**2**	
NPC + SOT	SD	0.76	6	0.38	3.00	0.75	5	0.56	4	0.82	7	0.35	2	0.33	1
	CV	3.71	7	1.77	3.00	3.24	6	2.05	4	3.07	5	1.45	2	1.37	1
	r	0.929	2	0.614	7.000	0.941	1	0.796	5	0.906	3	0.735	6	0.820	4
	Mean of sub-ranks		4.67		4.33*		4.33*		4.33*		5.00		3.33		2.00
	BK ranking	**4**		**3**		**3**		**3**		**5**		**2**		**1**	
NC + S	SD	1.28	4	1.29	5.00	1.49	6	0.91	1	2.67	7	1.22	3	1.14	2
	CV	6.61	5	6.23	4.00	6.96	6	3.44	1	10.70	7	5.30	3	4.94	2
	r	0.824	7	0.912	6.000	0.957	3	0.984	2	0.991	1	0.942	5	0.946	4
	Mean of sub-ranks		5.33		5.00*		5.00*		1.33		5.00*		3.67		2.67
	BK ranking	**5**		**4**		**4**		**1**		**4**		**3**		**2**	
NC + SOT	SD	0.84	6	0.45	3.00	0.83	5	0.51	4	0.86	7	0.44	2	0.31	1
	CV	4.12	7	2.13	4.00	3.61	6	1.90	3	3.21	5	1.80	2	1.30	1
	r	0.926	1	0.561	7.000	0.922	2	0.657	6	0.911	3	0.783	5	0.849	4
	Mean of sub-ranks		4.67*		4.67*		4.33*		4.33*		5.00		3.00		2.00
	BK ranking	**4**		**4**		**3**		**3**		**5**		**2**		**1**	
PC + KG	SD	1.03	5	0.27	2	1.45	7	0.57	4	1.04	6	0.32	3	0.27	1
	CV	5.16	6	1.28	2	6.44	7	2.13	4	4.02	5	1.32	3	1.12	1
	r	0.991	1	0.819	6	0.982	2	0.921	4	0.969	3	0.885	5	0.743	7
	Mean of sub-ranks		4.00*		3.33		5.33		4.00*		4.67		3.67		3.00
	BK ranking	**4**		**2**		**6**		**4**		**5**		**3**		**1**	

SD—standard deviation, CV—coefficient of variation, r—coefficient of correlation.

* Same ranking for a given dataset

Based on comparative ΔCt method, the highest stability in the whole dataset showed *AfUBC1* (SD = 0.723), *AfUBC2* (SD = 0.752) and *AfGAPDH1* (SD = 0.790), while the lowest *AfGAPDH2* and *AfTUA1* with SD of 0.915 and 1.065, respectively (Table 6). In most data subsets, two paralogs of *UBC* were ranked as any of the top three accompanied either by *AfGAPDH1* or *AfTBP2*. Only in the data subset comprising PD caryopses treated with plant growth regulators (PC+KG), *AfUBC2* was ranked quite low (SD = 0.755; 5^th^ place) and the three most stable genes were *AfUBC1* (SD = 0.0,687), *AfTBP2* (SD = 0.690) and *AfGAPDH1* (SD = 0.693).

**Table 6:** Ranking of candidate genes according to expression stability values (SV) calculated by ΔCt method. For dataset symbols, refer to Table 3.

Sample set	Candidate reference gene													
	*AfEF1a*		*AfGAPDH1*		*AfGAPDH2*		*AfTBP2*		*AfTUA1*		*AfUBC1*		*AfUBC2*	
	SV	Rank	SV	Rank	SV	Rank	SV	Rank	SV	Rank	SV	Rank	SV	Rank
Total	0.879	5	0.79	3	0.915	6	0.851	4	1.065	7	0.723	1	0.752	2
NPC	0.854	5	0.79	4	0.857	6	0.715	2	0.92	7	0.698	1	0.734	3
NPC+SOT	0.744	6	0.673	3	0.711	5	0.693	4	0.744	7	0.651	2	0.599	1
NC+S	1.244	6	0.916	4	0.991	5	0.881	3	1.784	7	0.826	1	0.844	2
NC+SOT	0.813	6	0.736	4	0.782	5	0.728	3	0.834	7	0.706	2	0.672	1
PC+KG	0.705	4	0.693	3	0.935	7	0.690	2	0.794	6	0.682	1	0.755	5

### Relative expression of *AfVP1* gene

**Fig 4:**
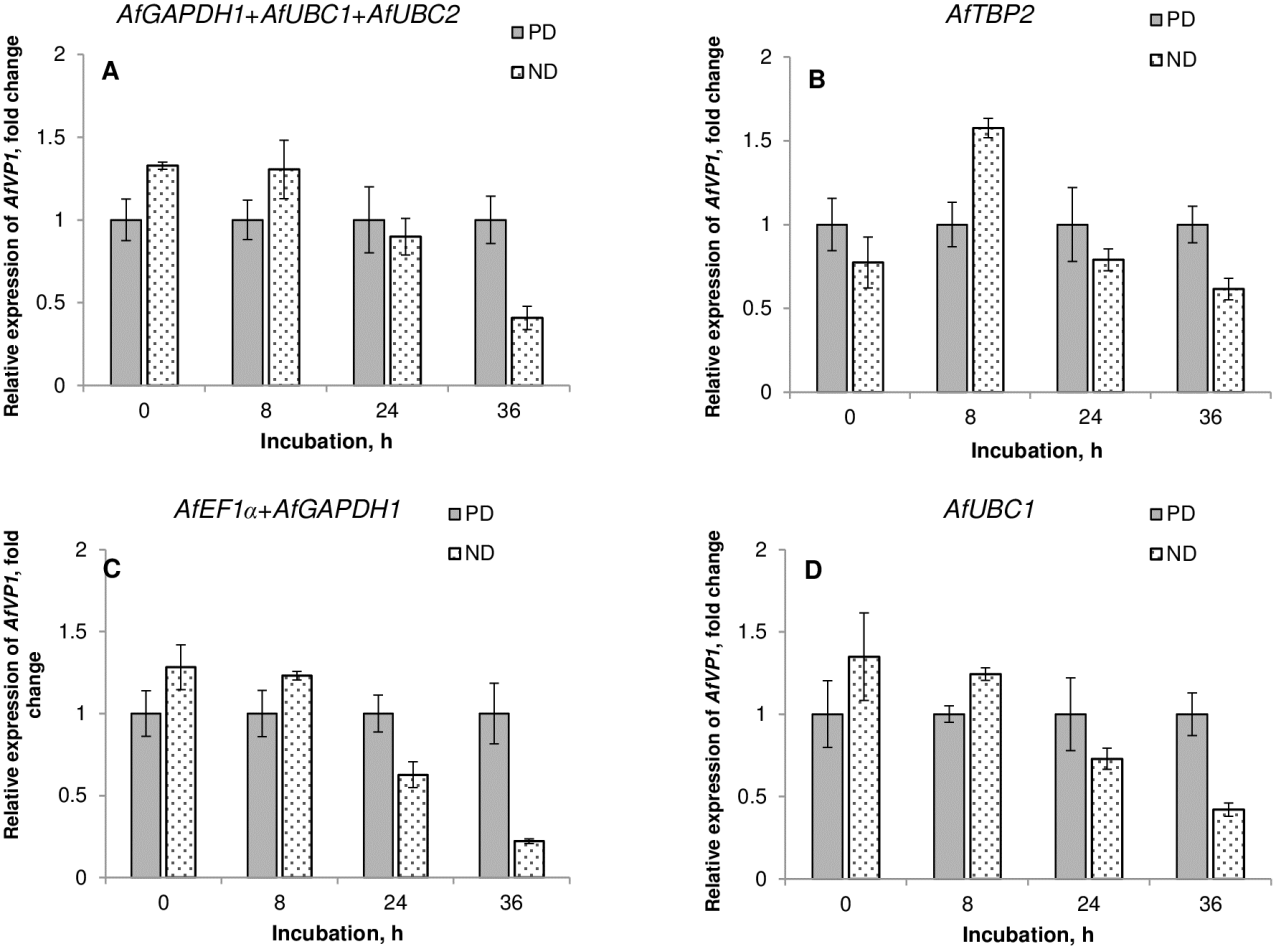
Relative expression of *AfVP1* in ND versus PD *A*. *fatua* caryopses during incubation on water at 20°C (NPC sample group). Different normalization factors were used, optimal according to geNorm (A), NormFinder (B, C), BestKeeper (D) and ΔCt method (D). The fold changes indicate the expression levels of *AfVP1* gene in non-dormant caryopses relative to calibrator (PD caryopses) with assumed value of 1 at each time-point.

There was no absolute agreement between the four statistical algorithms as to which of candidate reference genes or their combination could be considered as the most appropriate for qRT-PCR analyses in *A*. *fatua* dormancy/germination studies. Therefore, for NPC data subset, we compared the performance of single and multiple normalization factors suggested as optimal by each stability assessment method (Fig 4). The relative expression levels of the target gene (*AfVP1*) were calculated at distinct time-points during imbibition of ND in parallel with PD caryopses. The overall observation of *AfVP1* expression profiles revealed that there was not much difference in its transcript abundance between dry ND and PD caryopses. It was also not much regulated during the beginning of ND caryopses germination process up to 24 h of incubation. Only 2 hours before radicle protrusion through coleorhiza (36 h of incubation), there was observed a downregulation of *AfVP1* by about twofold. The results obtained through normalization using candidate reference genes indicated as the most suitable by GeNorm (combination of three genes: *AfGAPDH1*, *AfUBC1*, *AfUBC2*) (Fig 4A), NormFinder (combination of two genes: *AfEF1a*, *AfGAPDH1*) (Fig 4C) as well as BestKeeper and ΔCt method (single gene: *AfUBC1*) (Fig 4D) gave generally similar patterns of *AfVP1* gene expression. However, if the normalization was conducted with the *AfTBP*, the most stable single gene according to NormFinder, the expression profile of *AfVP1* was quite different (Fig 4B). In that case, there was unexpectedly higher expression of *AfVP1* in ND caryopses, as compared with PD ones, at the beginning of incubation (8h) and only slightly reduced shortly before the completion of germination (36h).

## Discussion

Comparative studies of the dormancy-related gene expression in *A*. *fatua* caryopses of different dormancy status, employing a powerful method of RT-qPCR, may provide insights into molecular mechanisms of the dormancy induction, maintenance and release as well as germination process per se. Therefore, several experiments were planned and performed to obtain reliable results which would facilitate further work on the expression profiles of the genes of interest involved in those physiological phenomena in *A*. *fatua*. Although there are several publications dedicated to reference genes used in seed biology studies [40, 47, 48, 58, 59, 60], to our knowledge, this is the first report concerning reference gene selection and validation for that specific developmental stage of *A*. *fatua* life cycle comprising dormant and germinating caryopses.

Since, among other factors, dormancy mechanisms contribute to persistence of the soil seed banks [61], it seems vital to reveal its mechanisms in a serious weed species like wild oat in order to enable improvements in integrated weed-control strategies. Taking this practical aspect into account, the investigations were focused on the issue of hormonal regulation of primary and secondary dormancy in natural populations of *A*. *fatua*, thus field-collected batches of caryopses were used in the studies.

If biologically significant results are to be obtained from RT-qPCR gene expression analyses, the raw data must be normalized to alleviate the influence of technical variation which is usually accomplished by the parallel quantification of endogenous reference genes [33, 34, 52, 62]. For a good reference gene it is expected that its expression variation should not arise from the regulation by experimental conditions, but reflect the errors produced by the sample processing steps. Therefore, in the present study, a SYBR green-based RT-qPCR assay was carried out on several candidate reference genes (*AfEF1α*, *AfGAPDH1*, *AfGAPDH2*, *AfTBP2*, *AfTUA1*, *AfUBC1 and AfUBC2*) using different samples of *A*. *fatua* in order to identify the most stable ones. The stability assessments were conducted on the whole dataset in order to compare different plant organs: seeds (caryopses) v. seedlings (vegetative organs/tissues) and also in data subsets which matched different experimental setups focused on: i. the germination of ND caryopses up to seedling stage (NC+S) or in comparison to PD caryopses (NPC); ii. the influence of supraoptimal temperature on ND caryopses (NC+SOT) also in comparison to PD caryopses (NPC+SOT); iii. the release of PD by KAR_1_ or GA_3_ (PC+KG) (Table 3, S1 Table).

Vast experimental evidence leads to the conclusion that there are no genes that are constantly expressed throughout the different stages in the plant’s lifecycle [41]. The levels of expression and thus particular transcripts abundance in different organs and developmental stages may substantially differ, which was also observed when comparing caryopses and seedlings of *A*. *fatua* (Fig 1). The expression of four out of seven candidate reference genes (*AfGAPDH1*, *AfTUA1*, *AfUBC1* and *AfUBC2*) was induced after germination. In our experimental system, comprising dry and imbibed caryopses additional factor might have affected gene expression and thus hinder the finding of an appropriate reference gene. That is the dry-to-hydrated (inactive-to-active) state transition being a part of the germination process [63]. We did observe a fluctuating expression levels of some candidate reference genes i.e. *AfEF1*α, *AfGAPDH2*, *AfTBP2* and *AfTUA1* which could be specifically attributed to the change in the moisture content and metabolism activation in *A*. *fatua* caryopses (Fig 1). Similar concerns were addressed by Chen et al. [58] who suggested that most house-keeping genes, traditionally chosen as reference genes for normalization in qPCR analyses, were influenced by the “hydration bias”.

The results of four widely accepted statistical algorithms employed for gene stability evaluation in *A*. *fatua*, did not distinguish one universal most stable gene to serve as an internal control in all of our experimental layouts. However, starting with a whole dataset, whichever the method used, at least one of two homologs of *AfUBC* with either *AfGAPDH1* or *AfTBP2* were appointed as highly (1^st^ to 3^rd^ rank) stable genes (Fig 2, Tables 4–6). In comparison, *AfGAPDH2* and *AfTUA1* were indicated as least stable, occupying from 5^th^ to 7^th^ place in the rankings. In most data subsets, a similar tendency in the stability of candidate genes was observed, although some discrepancies in results returned by different algorithms appeared. The differences in stability estimation due to the applied methods have been also observed elsewhere [10, 59, 62, 64, 65] and mainly attributed to differences in the type of input data (raw v. normalized Cq) and the parameters upon which the rankings are based. In several studies, the problem of the disagreement between the four methods has been solved by ranking the candidate reference genes either according to the geometric mean of the ranking numbers calculated e.g. by a web-based tool RefFinder [10, 57, 66] or the consensus reached with a cross-entropy Monte Carlo algorithm offered by RankAggreg, an R package for weighted rank aggregation [67, 68]. Robledo et al. [65] criticized the motion of selecting the best reference gene(s) due to such consensus rankings as having no biological meaning and recommended, instead, relying rather on assessment of the two most important and complementary issues i.e. absence of intergroup variation and correlation between reference genes provided by NormFinder supported by descriptive statistics from BestKeeper (SD, CV and r) if the four methods disagree. According to Kozera and Rapacz [33], it is advisable to use at least a pair of genes responsible for distant functions, because of a very little chance for a common regulation of their expression. Others advise to use at least three reference genes in order to achieve best results [69, 70]. As shown by the pairwise variation analysis (Fig 3) and further confirmed by the validation experiment in which the target *AfVP1* gene expression was assessed (Fig 4, S2 Fig), in our experimental conditions also two or three reference genes of high expression stability, performed better than a single gene.

The selection of an appropriate reference gene for normalization may have a great impact on the conclusions drawn from the experiment on biological significance of target gene expression data. Since there was no absolute agreement between different algorithms as to how many and which reference genes would be most suitable for RT-qPCR normalization, different possibilities were checked by normalizing raw Cq values of *AfVP1* gene (Fig 4, S2 Fig). Our data show that the combined use of at least two out of three the most stable reference genes (*AfGAPDH1*, *AfUBC1* and *AfUBC2*) can provide an optimal normalizing factor for qRT-PCR analyses comprising ND and/or PD caryopses. *UBC* belongs to the group of traditional reference genes and has been often reported as having a high expression stability [41, 71, 72]. It was the only classic HKG found in a set of highly stable genes in *Arabidopsis thaliana* and tomato seeds [37, 47]. The *UBC* gene was ranked the third among 21 genes from the results of overall ranking of the best reference genes for all samples including seeds of *Euphorbia esula* [60]. *GAPDH* gene was also indicated as one the most stable genes in herbicide-resistant *A*. *fatua* populations treated with different herbicides [10]. Taking into account a very high sequence similarity (99.3%) (Table 2) between the NGS sequenced transcript Unigene36693 designed *AfGAPDH1* and *GAPDH* partial cds (KT153027) it may be assumed that they represent the same gene homolog. In comparison, the other *GAPDH* homolog identified through NGS sequencing in wild oat, Unigene36694 (*AfGAPDH2*) was quite unstable in our experimental system (Fig 1, Tables 4–6). This observation may reflect the disagreement in reports concerning *GAPDH* gene expression stability. For example, *GAPDH* was recommended as the appropriate reference gene during seed development in tung tree [48] or under different germination time points in *Suaeda aralocaspica* [46] as well as in combination with *MDH* (*malate dehydrogenase*) for gene expression analysis in pulp and seed samples of *Theobroma grandiflorum* [59], but the results in Euphorbia showed that the levels of transcripts from *GAPDH1* and *GAPDH2* were very unstable in buds, seeds, and various organs [60]. As pointed by Saraiva et al. [73] such contrasting results between different species or even within the same species not necessarily should be attributed to differential expression behavior in response to distinct treatments, they as well might be caused by primer heterogeneity from different studies that could amplify different members of a gene family and thereby presenting a differential expression profile.

## Conclusions

From our findings it can be concluded that the normalization factor calculated as a geometric mean of Cq values of two *UBC* homologs accompanied by *AfGAPDH1* would be optimal for RT-qPCR results normalization in the experiments comprising *A*. *fatua* caryopses of different dormancy status. This conclusion remains in agreement with the approach proposed by Vandesompele et al. [52] and widely considered as the most appropriate in gene expression studies. Based on the obtained results it would be advisable to use *AfUBC1*, *AfUBC2* and *AfGAPDH1* as reference genes also in further studies on molecular basis of seed dormancy induction, maintenance, and release in *A*. *fatua* caryopses on condition of prior validation.

## Supporting information

S1 Fig Melting curves of *A*. *fatua* candidate reference and target genes. (TIFF)

S2 Fig Relative expression levels of *AfVP1* in PD and ND *A*. *fatua* caryopses during incubation on water at 20°C (NPC sample group). Most stable candidate reference genes (*AfGAPDHA1*, *AfUBC1* and *AfUBC2*), single or in combinations were used as normalization factors.
(TIFF)

S1 Table Description of sample groups and germination experimental conditions. (TIFF)

S2 Table Identification of transcripts in *Avena fatua* unigene assembly using *Poaceae* transcripts as queries. (XLSX)

S3 Table Coding sequences of *Avena fatua* L. candidate reference and target genes found in NGS RefSeq unigene dataset by Geneious software. Locations of qRT-PCR primers marked in colour (forward in blue, reverse in orange).
(DOCX)

S1 File Dataset of *de novo* assembled *Avena fatua* L. unigene sequences. RNA-seq library constructed using a pooled sample of RNA isolated from caryopses and seedlings was sequenced using an Illumina HiSeq2000 genome analyzer at customer service of BGI-Tech Solutions Co., Ltd., (Hong Kong, China).
(7Z) Click here for additional data file.
