# Supplementary figures and images for: Reference gene selection for molecular studies of dormancy in wild oat (Avena fatua L.) caryopses by RT-qPCR method
Source: PLoS One. 2018 Feb 1;13(2):e0192343. doi: 10.1371/journal.pone.0192343 (PMC5794185; doi:10.1371/journal.pone.0192343)

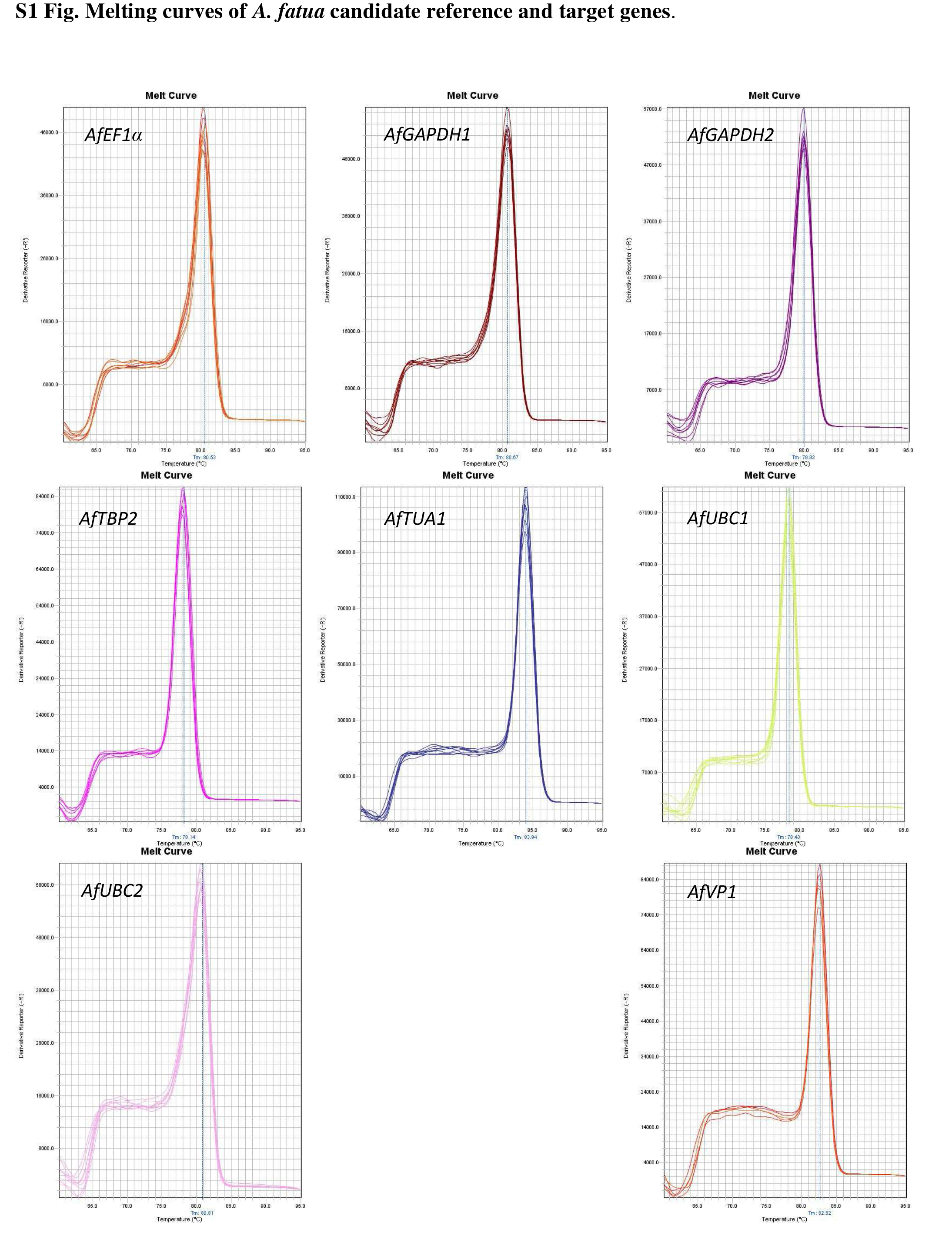

Supplement: S1 Fig — (TIFF) [file pone.0192343.s001.tiff]

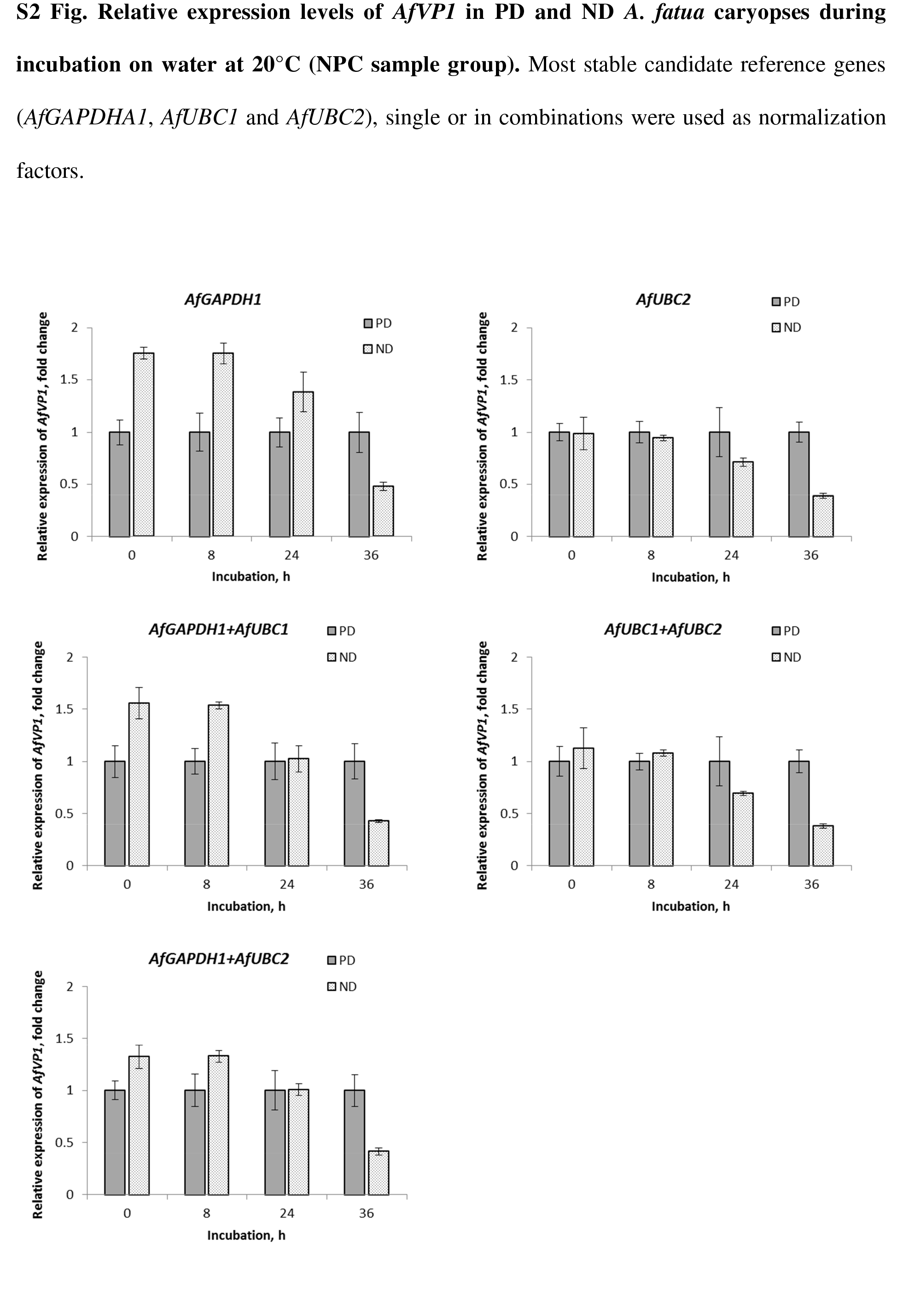

Supplement: S2 Fig — Most stable candidate reference genes (AfGAPDHA1, AfUBC1 and AfUBC2), single or in combinations were used as normalization factors. (TIFF) [file pone.0192343.s002.tiff]

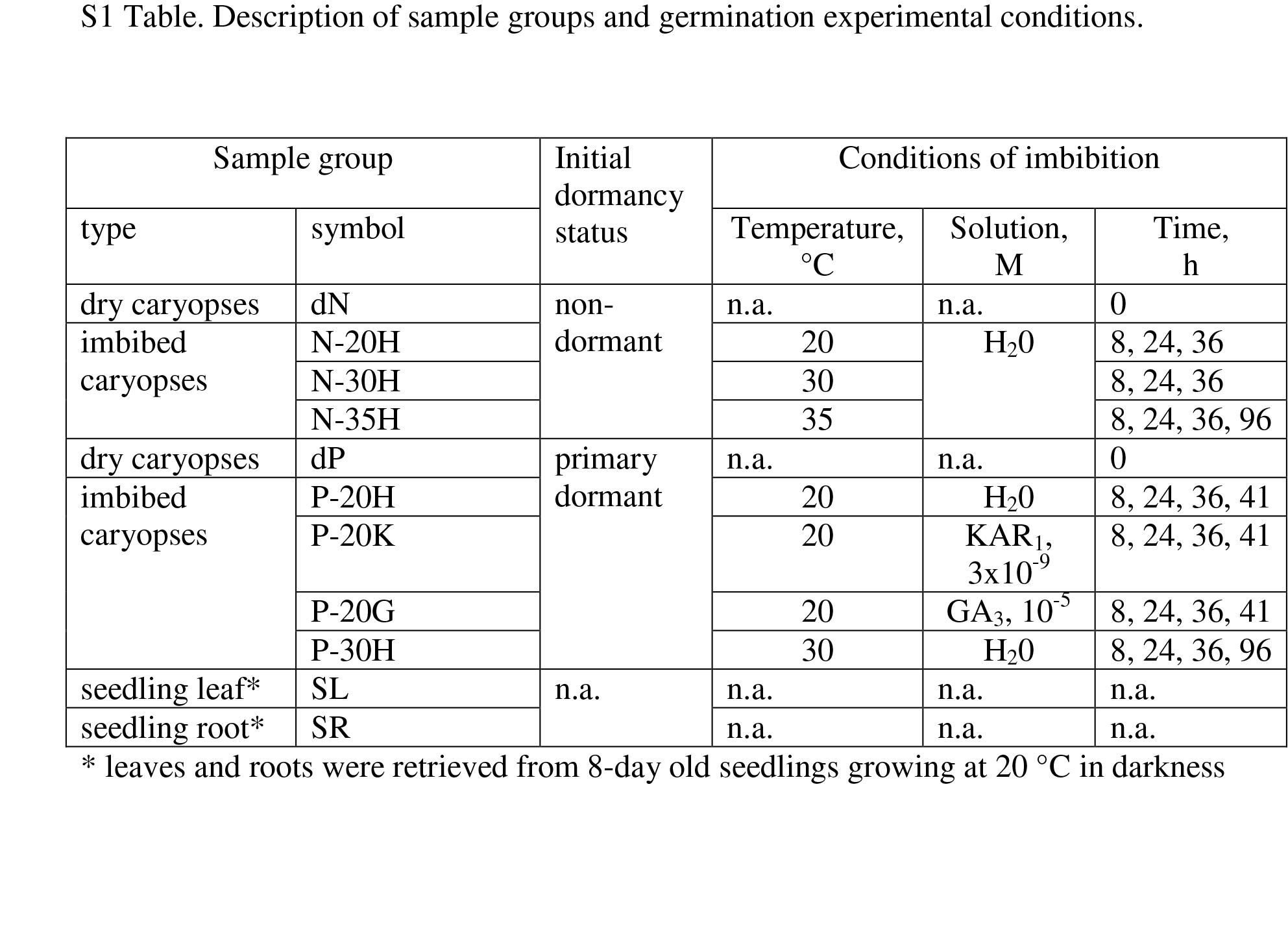

Supplement: S1 Table — (TIFF) [file pone.0192343.s003.tiff]
