# Supplementary material for: Reference gene selection for molecular studies of dormancy in wild oat (Avena fatua L.) caryopses by RT-qPCR method
Source: PLoS One. 2018 Feb 1;13(2):e0192343. doi: 10.1371/journal.pone.0192343 (PMC5794185; doi:10.1371/journal.pone.0192343)
Supplement: S3 Table — Locations of qRT-PCR primers marked in colour (forward in blue, reverse in orange). (DOCX) [file pone.0192343.s005.docx]

**S3 Table. Transcripts of *Avena fatua* L. candidate reference and target genes found in NGS RefSeq unigene dataset by Geneious software.** Locations of qRT-PCR primers marked in colour (forward in blue, reverse in orange). Homologous sequences that have not been subjected to RT-qPCR analyses are marked with an asterix (*).

***AfEF1α***

>CL3197.Contig1

ATGGGTAAGGAGAAGATTCACATCAGCATTGTGGTCATTGGCCATGTCGACTCTGGCAAGTCGACAACCACTGGCCACCTCATCTACAAGCTTGGAGGCATCGACAAGCGTGTGATCGAGAGGTTCGAGAAGGAAGCTGCTGAGATGAACAAGAGGTCCTTCAAGTACGCGTGGGTGCTGGACAAGCTCAAGGCTGAGCGTGAGAGGGGTATCACCATCGATATTGCCCTCTGGAAGTTCGAGACTACCAAGTACTCCTGCACTGTCATCGATGCTCCTGGACACCGTGACTTCATCAAGAACATGATCACCGGTACCTCCCAGGCCGATTGTGCTGTGCTTATCATTGACTCCACCACTGGTGGTTTTGAGGCTGGTATCTCCAAGGATGGCCAGACCCGTGAGCACGCTCTCCTTGCTTTCACTCTTGGAGTGAAGCAGATGATCTGCTGCTGCAACAAGATGGATGCCACCACCCCCAAGTACTCCAAGGGCCGTTTTGAGGAAATTGTCAAGGAAGTTGGCTCCTACTTGAAGAAGGTGGGCTACAACCCTGACAAGGTCCCATTTGTCCCCATCTCTGGATTTGAGGGTGACAACATGATTGAGAGGTCCAGCAACCTTGACTGGTACAAGGGCCCCACCCTTCTTGAGGCGCTTGACCAGATCAACGAGCCCAAGAGACCCTCAGACAAGCCCCTCCGCCTTCCCCTTCAGGATGTCTACAAGATTGGTGGCATTGGAACTGTGCCAGTGGGGCGTGTTGAGACTGGAGTGATCAAGCCTGGTATGCTTGTCACCTTTGGCCCAACTGGGCTGACCACTGAGGTGAAGTCTGTGGAGATGCACCACGAGTCCATGCTGGAGGCTGTGCCAGGTGACAACGTTGGCTTCAACGTGAAGAACGTTGCTGTGAAGGATATCAAGCGTGGGTATGTCGCCTCCAATGCCAAGGACGACCCTGCCAAGGAGGCTGCCAACTTCGTTGCCCAGGTCATCATCATGAACCACCCTGGCCAGATCGGCAACGGCTACGCCCCAGTGCTGGACTGCCACACGTCCCACATCGCTGTCAAGTTCTCTGAGATCCAGACCAAGGTTGACAGGCGTTCTGGCAAGGAGATTGAGGCCTTCCCCAAGTTCCTCAAGAACGGTGATGCTGGCTTCGTGAAGATGATTCCCACCAAGCCCATGGTGGTGGAGACCTTCTCCCAGTACCCTCCCCTTGGCCGCTTTGCAGTGCGTGACATGAGGCAGACTGTTGCTGTTGGTGTTATCAAGAGCGTGGAGAAGAAGGAACCCACTGGCGCGAAGGTGACCAAGGCTGCTGCCAAGAAGAAATGA

***AfGAPDH1***

>Unigene36693

ATGGGCAAGATTAAGATCGGAATCAACGGTTTCGGAAGGATCGGGAGGCTCGTCGCCAGGGTCGCCCTCCAGAGCGACGATGTCGAGCTCGTCGCCGTCAACGACCCCTTCATCACCACCGAGTACATGACCTACATGTTCAAGTACGACACCGTGCACGGCCACTGGAAGCACAGCGACATCAAGCTCAAGAACGACAAGACTCTCCTCTTCGGCGAGAAGGCGGTTACTGTCTTTGGCGTCAGGAACCCTGAAGAAATCCCATGGGCTGAGGCTGGTGCCGACTATGTCGTTGAGTCCACCGGTGTATTCACTGACAAGGACAAGGCTGCTGCTCACTTGAAGGGTGGTGCCAAGAAGGTGGTCATTTCAGCCCCTAGCAAAGATGCCCCTATGTTTGTGGTTGGTGTCAACGAGGACAAGTACACTTCTGATGTTAACATTGTCTCAAACGCTAGCTGCACAACAAACTGTCTTGCTCCCCTTGCCAAGATCATTAATGACAACTTCGGTATTGTTGAGGGTCTGATGACCACTGTTCATGCCATCACTGCCACTCAGAAGACTGTTGATGGTCCCTCAAGCAAGGACTGGAGAGGTGGGAGAGCTGCTAGCTTCAACATCATTCCCAGCAGCACTGGCGCTGCCAAGGCTGTTGGTAAGGTTCTTCCTGAGTTGAACGGCAAGCTTACCGGTATGTCTTTCCGGGTACCCACTGTGGATGTGTCAGTTGTTGATCTCACCGTCAGAATCGAGAAGGCTGCATCATATGAGGACATCAAGAAGGCTATCAAGGCCGCATCTGAGGGAAACCTCAAGGGGATCATGGGCTTTGTTGAGGAAGATTTGGTCTCCACCGACTTCATTGGCGACAGCAGGTCGAGCATCTTTGACGCCAAGGCTGGAATTGCTCTTAACGACAACTTCGTCAAGCTTATCTCGTGGTACGACAATGAGTGGGGTTACAGCAACCGCGTCGTCGACTTGATCCGCCACATGGCCAAGACTCAGTAG

***AfGAPDH2***

>Unigene36695

ATGGGCAAGATTAAGATCGGAATCAACGGTTTCGGAAGGATCGGGAGGCTCGTCGCCAGGGTCGCCCTCCAGAGCGACGATGTCGAGCTCGTCGCCGTCAACGACCCCTTCATCACCACCGACTACATGACATACATGTTCAAGTATGACACTGTTCACGGACAGTGGAAGCACCATGAGGTTAAGGTCAAGGACGCCAAGACCCTTCTCTTCGGCGAGAAGGAGGTTGCTGTCTTTGGCTGCAGGAACCCTGAGGAGATCCCATGGGCCTCTGCTGGTGCTGACTACGTTGTTGAGTCCACCGGTGTTTTCACTGACAAGGACAAGGCTGCAGCTCACATCAAGGGTGGTGCCAAGAAGGTCATCATTTCTGCTCCCAGCAAGGACGCTCCCATGTTCGTCATGGGTGTTAACGAGAAGGAATACACCTCTGACATCACCATTGTCTCCAACGCTAGCTGCACCACTAACTGCCTTGCTCCCCTTGCTAAGGTTATCAATGACAAGTTTGGCATTGTTGAGGGTTTGATGACCACTGTTCACGCCATGACTGCAACCCAGAAGACTGTTGATGGTCCCTCAAGCAAGGACTGGAGAGGTGGAAGGGCTGCTAGCTTCAACATCATTCCCAGCAGCACTGGAGCTGCAAAGGCTGTTGGCAAGGTGCTCCCTGTCCTCAACGGAAAGTTGACAGGAATGGCCTTCCGTGTTCCAACTGTTGATGTTTCTGTTGTTGACCTGACCGTTAGACTTGAGAAGGCAGCCACCTATGAGCAGATCAAGGCTGCAATCAAGGAGGAGTCCGAGGGTAAGCTCAAGGGCATTCTGGGTTACGTTGATGAGGACCTTGTTTCCACTGACTTCCAGGGTGACAACAGGTCCAGCATCTTCGACGCCAAGGCTGGGATTGCTCTGAACGACAACTTTGTCAAGCTTGTGTCCTGGTACGACAACGAGTGGGGCTACAGCACCCGTGTAGTCGACCTGATCCGTCACATCCACGGCACCAAGTGA

***AfTBP1****

>CL3311.Contig2

CAGCCAGTGGACCTGTCCGTGCACCCCTCCGGCATCGTCCCCACGCTCCAGAATATTGTGTCCACGGTCAACTTGGACTGCAAATTGGACCTGAAAGCAATAGCTCTCCAGGCGCGCAATGCAGAATACAACCCCAAGCGTTTTGCTGCTGTTATCATGCGTATAAGAGAACCAAAAACTACTGCACTGATATTTGCATCAGGAAAAATGGTTTGCACTGGAGCAAAAAGTGAACAGCAGTCTAAACTCGCAGCTAGAAAGTATGCTCGTATAATCCAGAAGCTTAACTTTCCGGCGAAA

TTCAAGGACTTCAAGATCCAGAATATTGTTGCATCCTGTGATGTGAAATTCCCTATAAGGCTTGAAGGCCTGGCATATTCTCACGGCGCTTTCTCCAGTTACGAGCCAGAGCTATTTCCTGGTCTGATCTATCGGATGAGGCAGCCGAAGATTGTCCTGCTAATTTTTGTCTCGGGCAAGATTGTTCTGACTGGAGCGAAGGTGAGAGAACAGACATATACCGCCTTCGAGAACATATATCCCGTCCTCACAGAGTTCAGAAAAGTTCAG

***AfTBP2***

>CL4896.Contig1

GCAAAGAGTGAACAACAGTCCAAGCTTGCAGCAAGAAAGTATGCTCGTATTATTCAGAAACTTGGCTTTCCAGCCAAATTCAAGGACTTTAAGATTCAGAACATCGTTGCCTCTTGTGATGTCAAATTTCCAATTGGACTGGTGGGCCTTGCATATTCTCATGGTGCTTTCTCAAGTTATGAGCCAGAACTTTTTCCTGGCCTGATCTATCGAATGAAACAACCAAAGATTGTTCTTCTGATTTTTGTTTCGGGCAAGATTGTCTTGACCGGTGCAAAGGTGAGAGATGAGACATACACT

GCCTTTGAAAACATATATCCTGTACTCACAGAGTTCAGAAAAGTTCAGCAA

***AfTUA1***

>Unigene36141

TGCCTCGAGCACGGCATCCAGCAAGATGGCACCATGCCCAGTGATACCACAGTTGGGGTAGCACACGACGCGTTCAACACCTTCTTCAGTGAGACCGGCGCGGGCAAGCATGTGCCGAGGGCCATCTTCGTCGACCTTGAGCCCACCGTCATTGATGAGGTGCGCACCGGTGCCTACCGTCAGCTTTTCCACCCGGAGCAGCTCATCTCCGGGAAGGAGGATGCTGCTAACAACTTCGCCCGTGGCCACTACACTGTTGGAAAGGAGATCGTAGATCTATGTCTGGATCGTGTACGCAAGTTGGCAGACAATTGCACCGGGCTGCAGGGATTCCTGGTGTTCAATGCTGTCGGTGGTGGAACCGGATCAGGACTGGGTTCTCTTTTGTTGGAGCGCCTCTCAGTTGATTATGGAAAGAAATCTAAGCTTGGTTTCACCATTTACCCTTCCCCACAGGTCTCGACAGCTGTTGTAGAGCCCTACAACAGTGTTCTCTCCACTCACTCTTTGCTTGAGCACACCGACGTTGCAGTCCTCCTTGATAACGAAGCTATCTATGACATATGCCGGAGGTCCCTTGACATTGAGAGGCCAACTTACACCAACTTGAACAGGCTGATATCACAGATCATATCTTCACTTACCACCTCCCTAAGGTTTGATGGCGCCATCAATGTGGATGTCACCGAGTTCCAGACCAACCTTGTCCCATACCCACGTATCCATTTCATGCTTTCGTCGTATGCCCCTGTTATCTCTGCGGAGAAGGCTTACCATGAGCAGCTCTCAGTGCCTGAAATCACCAACGCTGTATTTGAGCCCTCAAGCATGATGGCCAAGTGTGATCCTAGGCACGGGAAATACATGGCTTGCTGCTTGATGTACCGTGGTGATGTTGTCCCCAAGGATGTCAATGCTGCAGTTGCGACCATCAAAACCAAGAGAACTGTCCAGTTCGTCGACTGGTGCCCTACTGGATTCAAGTGCGGTATCAACTACCAACCACCTTCAGTTGTCCCCGGAGGCGACCTGGCAAAGGTTCAGCGGGCCGTGTGCATGATCAGCAACAACACTGCTGTTGCTGAGGTATTCTCGCGCATCGACCACAAGTTCGACTTGATGTACGCCAAGCGTGCATTCGTGCACTGGTATGTCGGCGAGGGTATGGAAGAAGGTGAGTTCTCGGAAGCCCGTGAGGACTTGGCTGCCCTTGAGAAGGACTACGAGGAAGTCGGCGCTGAAGGCGCAGACGACGAGGGTGACGAGGGGGATGACTATTAA

***AfTUA2****

>Unigene2539

CAGGGTTTCCTTGTCTTCAATGCTGTTGGAGGTGGAACTGGCTCTGGCCTTGGTTCTCTCCTCCTTGAGAGGCTCTCTGTTGATTATGGAAAGAAGTCCAAGCTTGGGTTCACTGTGTACCCATCGCCTCAGGTCTCCACCTCTGTTGTTGAGCCATACAACAGTGTCCTGTCCACCCACTCCCTCCTGGAGCACACTGATGTGGCTGTTCTTCTCGACAATGAGGCCATCTATGATATATGCCGCCGCTCCCTTGATATTGAGCGCCCAACCTACACCAACCTCAACAGGCTTGTATCTCAGGTCATATCATCATTGACTGCTTCCCTGAGGTTTGATGGTGCTCTGAATGTGGATGTCAATGAGTTCCAGACCAATCTGGTGCCCTACCCAAGGATCCACTTCATGCTTTCCTCCTATGCCCCAGTTATCTCAGCTGAGAAGGCTTACCACGAGCAGCTCTCTGTTGCTGAGATCACCAACAGTGCCTTCGAGCCTTCCTCCATGATGGCCAAGTGTGACCCCCGCCACGGCAAGTACATGGCCTGCTGTCTGATGTACCGTGGTGATGTCGTGCCCAAGGACGTGAACGCTGCTGTGGCTACCATCAAGACCAAGCGCACCATCCAGTTCGTGGACTGGTGCCCCACTGGCTTCAAGTGCGGTATCAACTACCAGCCACCCAGCGTTGTCCCTGGTGGTGACCTTGCCAAGGTCCAGAGGGCTGTGTGCATGATCTCCAACTCCACCAGTGTTGTTGAGGTCTTCTCCCGCATCGACCACAAGTTTGACCTGATGTACGCCAAGCGTGCCTTCGTCCACTGGTACGTGGGTGAGGGTATGGAGGAGGGAGAGTTCTCTGAGGCCCGTGAGGATCTTGCTGCCCTGGAGAAGGACTACGAAGAAGTTGGTGCTGAGTTCGACGAGGGTGAGGATGGTGATGAGGGTGATGAGTAC

***AfUBC1***

>Unigene2728

ATGGCGTCCAAGCGGATCCTCAAGGAGCTCAAGGACCTGCAGCGGGATCCCCCCACCTCCTGCAGCGCAGGCCCTGTGGCAGAAGATATGTTCCACTGGCAAGCAACGATTATGGGTCCAACCGAAAGCCCATATGCCGGTGGCGTCTTTTTGGTTACTATCCACTTCCCACCGGACTACCCATTTAAGCCACCCAAGGTCGCATTCAAGACAAAGGTTTTCCATCCAAATATCAACAGTAACGGGAGCATATGTCTTGATATCTTGAAGGAGCAATGGAGCCCTGCATTGACAGTTTCCAAGGTACTCCTTTCAATCTGTTCTCTGCTAACGGACCCAAACCCCGACGACCCGTTGGTTCCGGAGATTGCACACATGTACAAGAGCGACCGTGTGAAGTATGAGTCCACCGCGAGGAGCTGGACCCAAAAGTACGCGATGGGCTGA

***AfUBC2***

>CL162.contig2

ATGGCGGCTGTGGGAAAGTTCAACCGGAGCAACCCGGCGGTGAAGCGGATCCTGCAGGAGGTGAAGGAGATGCAATCCAATCCCTCCCCAGACTTCATGGCCCTGCCCCTCGAGGAGGACATTTTCGAGTGGCAATTTGCTATCATGGGGCCACGAGACAGCGAGTTTGAGGGAGGAATCTACCATGGGAGAATCCAGTTGCCCTCTGACTACCCGTTCAAGCCACCATCCTTCATGCTCCTCACGCCGAGTGGAAGATTTGAGATTCAGAAGAAGATATGTTTGAGCATATCAAACTACCACCCTGAGCACTGGCAGCCATCATGGAGTGTGCGCACAGCACTAGTAGCGTTGATTGCATTCATGCCAACAAATCCTGGCGGGGCATTGGGTTCACTGGACTACAAAAAGGAAGATAGACGAGCACTGGCTATCAAATCACGTGAAACACCACCAAAATTTGGCTCCCCAGAACGCCAAAGAGTGATTGATGAGATCCATGAGCAAATGCTCAGTAAAGCTCCAGCTGTTCCTCAACTCCTGACAAATGGCTCTGACGAGGACACTAACAAGTTAACCCCACCAGACGTTTCTGGTGAAGACGTTTCTGGTGAACATGCTGACAATCCAGCTGAAGGTGCCAACACTTCTGGCTCTGCTAATACTGACCTTCCAAAGCCCGATTCAGAGTCAGAAGTTGCTGAGAACATTGTTGAAGCTCAGTCAGATGTAATCCCCAGGGACAGCATTCCGAGAGTTGTTGCGGCACCACAGAATCCTGTTGTTACAATTCAGAAGCCAAAGCATGACAGATTGTTGACGTTGGCTGCATTCGGGCTGACTCTTGCTATTATGGCCCTTGTGATAAAGAAGTTCTTCAAGATCAATGGTCTGGCTGGTTACATTGAGGGAAAGTTTTAG

***AfVP1***

>CL7717.Contig1

ATGGACGCCTCCGCCGGCTCCTCGCCGCCGCCGCACTCGCAGGAGAACCCGCCCAAGCACGGTGGAGGCCGCGGGAAGGCCCCTGCGGGGGAGATCCGGAAGGGAGAGGCGGCCACGGCGGATGACTTTATGTTCGCGGAAGATACCTTCCCGTCCCTCCCGGATTTCCCTTGCCTCTCCTCCCCGTCAAGCTCCACCTTCTCCTCCTCATCCTCCTCCAACTCATCCAGCACCCTCGCCGCCGCGGGACGCGGGCGTGGCCGTTGTCGCGGAGGCGCGCCCGAAGGCGCCCTCGGGGAGCCCTCCGATCCTGCTGCCGCGGGGGAGGATGACGTGCTCGACGACATCGACGAGCTGCTCAACTTCGCCACGCTCTCCGACTCCATGCCCTGGGAGGACGAGCCGCTCTTCCCCGACGACGTTGGCATGATGATAGAGGACGCCATCTCCCACCAGCCGCCTGCTGCGGGCCACCGCGGTGCGAGGAACGCTGCATCGTCGGAGGCGGCTGGTGGTGGTGGTGGACAGGATTCCTCGTCGGCGGCGGACGACCTGCCGCGGTTCTTCATGGAGTGGCTGACGAACAACCGCGACTGCATCTCCGCCGAGGACCTCCGCAACATCCGCCTCCGCCGCTCGACCATCGAGGCCGCGGCGGCGCGGCTCGGTGGAGGGCGGCAGGGCACCATGCAGCTGCTCAAGCTCATCCTCACATGGGTGCAGAACCACCATCTGCAGAAGAAGCGCGCCCGCGTCGACGACGAGCTCCCCAGCCCCGGCGCAAACCCGGGTTACGAGTTCCCCGCGGAGACAGTTGCCCCCGCCACATCCTGGCTCATGCCCTACCAACAAGCTTATGGAAGAGAGGCGATCTACCCGAACGCCGCCGCCACCGGGCAGTACCCATTCCAGCAGGGCGGCAGCACGAGCAGCGTGGTGGTGAGCAGCCAGCCGTTCTCCCCGCCGGCGCCGGTGGCCGACATGCAGGCGGCGAACATGCCCTGGCCGCAGCAGTACGCGGCGTTCCCCGGCGCTGCGCCATACCCGATGCCGCCGCCGCAGCCGTTGGCGGCGGCCGGATTCGGCGTGTGCCCGCAGCCCTTGGCCGGGGTGAAGCCGTCGGCGAGCAAGGAGGCCCGGAAGAAGCGTATGGCGAGGCAGCGCCGCCTCTCCTGCCTGCAGCATCAGCGGAGCCAGCAGCTGAATCTGGGCCAGATCCAGAACGCCATGATCCATCCGCAGCAGGAGGTGCCGTTCTCTCCCCGCTCCGCGCACTCGGTGCCTGTCTCGCCGCCGTCGCCCGGCGGCTGGTGCGGGCTCTGGCCGCCGCCCTCCGTCCAAGTCCAGGGCCAGGGCCAACTCATGGTCCCGAATCCGCTGTCGACAAAGCCCAATTCCTCCTCGAGGCAGAAGGCGCAGAAACCCTCGCCGGACGCAGGAGCAAGACCGCCGTCGTCCGGCGCGCAGCAGGGTGCGAAGCCGGCGGCGGACAAGAATCTGCGGTTTCTGCTGCAGAAGGTGCTGAAGCAGAGCGACGTCGGCGCCCTCGGCCGCATCGTGCTCCCCAAAGAAGCGGAGACGCACCTGCCGGAGCTCAAGACGAGGGACGGCATCTCCATCCCCATGGAGGACATCGGCACCTCTCGGGTCTGGAGCATGCGGTACCGGTTTTGGCCTAACAACAAGAGCAGAATGTATCTCCTTGAGAACACTGGGGACTTTGTTCGGTCAAACGAGTTGCAGGAGGGCGACTTCATCGTAATTTACTCAGATGTCAAGTCGGGCAAATATCTGATACGTGGTGTGAAGGTAAGACCTCCCCAGGATCTAGCGAAGCAGAAGCATGGCAGTCTAGAGAAAGGCAGCACCTCAGATGCGATGGCGTGCGCTGAAGACGGTGGTGGCGCCGAGGCGGGCGGCTGCAAGGGGAAGTCTCCGCACGGCGTCAGGCGGTCTCGCCAGGAGGCTGCGTCCATGAACCAGATGACGGTGAGCATATGA
